# Differential impact of white matter hyperintensities on long-term outcomes in ischemic stroke patients with large artery atherosclerosis

**DOI:** 10.1371/journal.pone.0189611

**Published:** 2017-12-12

**Authors:** Minyoul Baik, Kyoungsub Kim, Joonsang Yoo, Hyeon Chang Kim, Seong Ho Jeong, Ki Hoon Kim, Hyung Jong Park, Young Dae Kim, Ji Hoe Heo, Hyo Suk Nam

**Affiliations:** 1 Department of Neurology, Yonsei University College of Medicine, Seoul, Republic of Korea; 2 Department of Neurology, Keimyung University School of Medicine, Daegu, Republic of Korea; 3 Department of Preventive Medicine, Yonsei University College of Medicine, Seoul, Republic of Korea; Shanghai Institute of Hypertension, CHINA

## Abstract

**Background:**

The presence of white matter hyperintensity (WMH) is related to poor long-term outcomes in stroke patients. However, the long-term outcome is unknown in patients with both large artery atherosclerosis (LAA) and WMH.

**Methods:**

We investigated the impact of WMH on long-term outcome in patients with LAA. Consecutive patients in a prospective stroke registry were included. Patients were followed for a median of 7.7 years (interquartile range, 5.6–9.7). The degree of WMH was assessed by Fazekas grade on fluid-attenuated inversion recovery images. Total WMH burden was calculated by summation of Fazekas scores in periventricular and deep white matter. Severe WMH was defined as total burden score ≥ 3.

**Results:**

Among 2529 patients, 639 patients (25.3%) were classified with the LAA subtype. After applying exclusion criteria, the data from 538 patients were analyzed. The mean patient age was 65.7 ± 10.3 years. Severe WMHs were found in 243 patients (45.2%). During follow-up, 200 patients (37.2%) died. Cox regression analysis showed that LAA patients with severe WMH had a 1.50-fold (95% CI, 1.12–2.00, p = 0.007) higher death rate compared to those without. In the older age group (≥65 years), Cox regression revealed that patients with severe WMH had a 1.75-fold (95% CI, 1.15–2.65, p = 0.008) higher 5-year death rate, whereas the younger age group did not have this association.

**Conclusion:**

The degree of WMH might be a surrogate marker for long-term outcome in patients with LAA. Atherosclerotic burdens in both small and large arteries might impact long-term prognosis in ischemic stroke patients.

## Introduction

White matter hyperintensity (WMH) is a small vessel disease defined as patchy or confluent periventricular and subcortical areas of higher signal intensity on magnetic resonance imaging (MRI) [1]. It is well known that WMH increases the risk of long-term mortality in the general population [2], young patients with ischemic stroke [3], and patients with systemic atherosclerotic diseases [4].

Among stroke subtypes, WMH is strongly linked to lacunar infarction as they share common small artery pathologies [5]. The small vessel disease is associated with microvascular pathogenesis including endothelial dysfunction and leakage of the blood-brain barrier. Recent studies also showed that WMH is also associated with large artery atherosclerosis (LAA) [6–9].

Considering the possible prognostic implication of WMH in combination with LAA, little is known about the impact of WMH on the long-term outcome in stroke patients with the LAA subtype. We hypothesized that ischemic stroke patients with LAA and WMH might have poor outcomes because they have both large and small artery diseases. Since WMH is a chronic process, we investigated long-term outcomes in patients with LAA according to the degree of WMH.

## Materials and methods

### Patients and evaluation

The study subjects were drawn from consecutive patients with acute ischemic stroke who had been registered in the prospective stroke registry from January 2001 to June 2007. During admission, all patients with cerebral infarction within 7 days after symptom onset were thoroughly investigated. Patients were evaluated with angiography, 12-lead electrocardiography, chest x-ray, lipid profile and standard blood tests. Transesophageal echocardiography, transthoracic echocardiography, heart CT, Holter monitoring and continuous EKG monitoring in stroke unit were also performed. Among the study patients, 388 (72.1%) patients underwent continuous EKG monitoring and 246 (45.7%) patients underwent echocardiography. More detailed information about etiologic evaluations of the patients was described in S1 Table.

Patients with acute ischemic stroke of LAA origin were included and those without MRI or fluid attenuation inversion recovery (FLAIR) image were excluded. LAA was defined when the patient had significant (≥50%) stenosis of the large cerebral artery relevant to the acute infarction, according to the Trial of ORG 10172 in Acute Stroke Treatment (TOAST) classification [10]. This study was approved by the Severance Hospital Institutional Review Board, Yonsei University Health System.

### Assessment of white matter hyperintensity

WMH was defined as supratentorial hyperintense lesions on FLAIR imaging according to the standards for reporting vascular changes on neuroimaging criteria [1]. MRI examinations were performed on either a 1.5T (Signa Horizon 1.5T, GE Medical System, Milwaukee, Wis; or Intera 1.5T, Philips Medical Systems, Best, Netherlands) or a 3.0T MRI system (Achieva 3.0T, Philips Medical Systems, Best, the Netherlands). MRI images were obtained parallel to the orbitomeatal line using the following parameters: time repetition (TR)/time echo (TE) = 2,600–6,500/42–70 ms, interslice gap = 2 mm, field of view (FOV) = 230×230 mm, slice thickness = 5 mm, six different directions of diffusion gradient (x, y, z, xy, yz, and zx), and two b values (0 and 1000) for diffusion-weighted imaging (DWI); TR/TE = 9,000/120 ms, FOV = 230×230 mm, pixel spacing = 0.449 mm/0.449 mm, and slice thickness = 5 mm for FLAIR imaging. When the patients underwent multiple MRI studies, the first MRI was used for white matter grading.

The degrees of both periventricular WMH and deep WMH were assessed using a 4-level ordinal scale (none, mild, moderate, or severe) based on the methodology of Fazekas [11]. Severe periventricular WMH and deep WMH was defined as Fazekas score ≥2. Total WMH burden was calculated by summation of the scores for periventricular WMH and deep WMH, and ranged between 0 and 6. The degree of WMH was dichotomized into no or mild (Fazekas score = 0 to 2) and severe (Fazekas score = 3 to 6) [12]. Two raters, who were blinded to the clinical findings, separately assessed WMH. Inter-rater reliability of determining the degree of WMH was excellent (the kappa values of periventricular WMH and deep WMH were 0.805 and 0.755, respectively).

### Functional outcome at 3 months and long-term mortality

Short-term functional outcomes at 3 months were determined based on the modified Rankin scale (mRS). Poor outcome was defined as a mRS score ≥ 3. Long-term mortality and causes of death were identified using death certificates from the Korean National Statistical Office. In Korea, by law, all deaths of Koreans must be reported to the National Statistical Office. For deaths not certified by physicians, any vague or missing item on the death certificate is clarified by the National Statistical Office via telephone [13]. Deaths among subjects from January 2001 to December 31, 2013 were confirmed by matching the information in the death records and identification numbers assigned to the subjects at birth [13]. The cause of death was coded according to the International Classification of Disease, 10^th^ revision. Cardiovascular death included fatal stroke (I60-64) and fatal ischemic heart disease caused by myocardial infarction (I21-23, I46).

### Statistical analyses

SPSS for Windows (version 21.0, SPSS Inc., Chicago, IL, USA) was used for statistical analysis. The Pearson Chi-square test was used to compare frequencies. For continuous variables, data distributions were examined for normality using the Kolmogorov-Smirnov test. If the data did not deviate from a normal distribution, the mean and standard deviation (SD) were calculated and independent sample t-tests were used for comparisons. For data that were not normally distributed, we reported descriptive statistics as the median and interquartile range (IQR) and compared them using the Kruskal-Wallis test. Independent predictors for poor outcome at 3 months were determined using the logistic regression analysis. The Kaplan-Meier analysis was used to estimate survival conditions and the log-rank test was used to compare rate estimates. The Cox proportional hazard regression analysis was performed to calculate crude and adjusted hazard ratios (HRs) with 95% confidence intervals (CIs). Variables with p <0.1 in the univariable analyses along with age and sex were entered in the Cox regression. Since older age is major determinant of severe WMH and long-term mortality, we tested the interaction effect between age and severe WMH at an alpha level of 0.15. To investigate the association between age and WMH, Spearman correlations were determined. And partial correlation coefficients after adjusting the values p <0.1 in univariable analyses were calculated. Steiger’s Z-test was used to compare these correlation coefficients.

## Results

### Study population

During the study period, 2529 cerebral infarction patients were registered in the stroke registry. 639 patients (25.3%) were categorized with the LAA subtype. After excluding 43 patients without MRI data and 58 patients without FLAIR imaging data, 538 patients were finally enrolled in the study. Severe WMH was found in 243 patients (45.2%). Patients with severe WMH were older (p <0.001), more frequently had hypertension (p = 0.004), and were less likely to be current smokers (p = 0.009) and men (p = 0.042) compared to those with no or mild WMH. Patients with severe WMH showed higher pulse pressure, and lower triglyceride level than those with no or mild WMH, whereas serum creatinine level was similar (Table 1).

**Table 1:** Demographic characteristics of study patients according to degree of white matter hyperintensity (WMH).

	Total	Severe WMH	No or mild WMH	*p*-value
	n = 538	n = 243	n = 295	
Age, years				
mean ± SD	65.7 ± 10.3	69.6 ± 8.3	62.5 ± 10.7	
median	67.0 [59.0–73.0]	69.0 [64.0–75.0]	63.0 [55.0–70.0]	<0.001
<65	233 (43.3)	68 (28.0)	165 (55.9)	
≥65	305 (56.7)	175 (72.0)	130 (44.1)	
Sex, men	361 (67.1)	152 (62.6)	209 (70.8)	0.042
Hypertension	416 (77.3)	202 (83.1)	214 (72.5)	0.004
Diabetes	221 (41.1)	98 (40.3)	123 (41.7)	0.749
Hyperlipidemia	68 (12.6)	28 (11.5)	40 (13.6)	0.479
Smoking	268 (49.8)	106 (43.6)	162 (54.9)	0.009
Pulse pressure (mmHg)	71.1 ± 20.6	73.3 ± 21.6	69.4 ± 19.6	0.034
Total cholesterol (mmol/L)	4.7 ± 1.0	4.7 ± 0.9	4.7 ± 1.0	0.725
Triglyceride (mmol/L)	1.5 ± 0.8	1.4 ± 0.7	1.6 ± 0.9	0.001
HDL (mmol/L)	1.1 ± 0.4	1.1 ± 0.4	1.1 ± 0.4	0.284
LDL (mmol/L)	2.8 ± 1.0	2.9 ± 1.0	2.8 ± 1.1	0.136
Serum creatinine (μmol/L)	1.0 ± 0.6	1.0 ± 0.6	1.0 ± 0.6	0.602
Initial NIHSS score	4.0 [2.0–8.25]	4.0 [2.0–9.0]	4.0 [2.0–8.0]	0.752
0–2	195 (36.2)	93 (38.3)	102 (34.6)	
3–5	133 (24.7)	56 (23.0)	77 (26.1)	
≥6	210 (39.0)	94 (38.7)	116 (39.3)	
Previous antithrombotics	40 (7.4)	18 (7.4)	22 (7.5)	0.982
Antiplatelet	37 (6.9)	16 (6.6)	21 (7.1)	0.807
Anticoagulant	4 (0.7)	3 (1.2)	1 (0.3)	0.332
Previous statin	24 (4.5)	11 (4.5)	13 (4.4)	0.947

Data are expressed as the mean ± SD, median [interquartile range], or number (%);

HDL, high density lipoprotein; LDL, Low density lipoprotein; NIHSS, National Institute of Health Stroke Scale.

### Functional outcome at 3 months

Patients with severe WMH showed similar poor outcome at 3 months compared to those with no or mild WMH (32.9 vs. 28.5%, p = 0.265). Univariable analyses showed that patients with hypertension, or higher initial NIHSS score were more likely to have poor functional outcomes at 3 months. Logistic regression analysis showed initial stroke severity was an independent predictor of poor outcome at 3 months. However, there was no difference in functional outcome at 3 months between patients with and without severe WMH (p = 0.525) (Table 2).

**Table 2:** Univariable and multivariable analyses for poor outcome at 3 months (mRS 3–6).

	Univariable		Multivariable	
	HR (95% CI)	*p*-value	HR (95% CI)	*p*-value
Age (years)				
<65	1			
≥65	1.20 (0.83–1.74)	0.342	1.12 (0.69–1.80)	0.649
Sex (men)	0.89 (0.60–1.31)	0.544	1.21 (0.75–1.97)	0.438
Hypertension	1.63 (1.02–2.61)	0.041	1.52 (0.86–2.68)	0.149
Diabetes	1.10 (0.76–1.60)	0.616		
Hyperlipidemia	1.11 (0.64–1.91)	0.720		
Smoking	0.94 (0.65–1.36)	0.751		
Pulse pressure (mmHg)	1.00 (1.00–1.01)	0.415		
Total cholesterol (mmol/L)	1.13 (0.93–1.37)	0.213		
Triglyceride (mmol/L)	0.78 (0.60–1.01)	0.057	0.82 (0.62–1.10)	0.190
HDL (mmol/L)	1.54 (0.95–2.48)	0.078	1.67 (0.95–2.93)	0.076
LDL (mmol/L)	1.03 (0.86–1.23)	0.789		
Serum creatinine (μmol/L)	0.96 (0.69–1.33)	0.791		
Initial NIHSS score				
0–2	1		1	
3–5	6.02 (2.75–13.17)	<0.001	6.08 (2.75–13.48)	<0.001
≥6	30.39 (14.74–62.66)	<0.001	30.95 (14.82–64.66)	<0.001
Previous antithrombotics	1.11 (0.56–2.20)	0.773		
Previous statin	1.15 (0.48–2.74)	0.756		
Severe WMH	1.23 (0.85–1.78)	0.265	1.17 (0.73–1.86)	0.525

Data are expressed as the hazard ratio (95% CI);

HDL, high density lipoprotein; LDL, Low density lipoprotein; NIHSS, National Institute of Health Stroke Scale; WMH, white matter hyperintensity.

### Cumulative death rates

**Fig 1:**
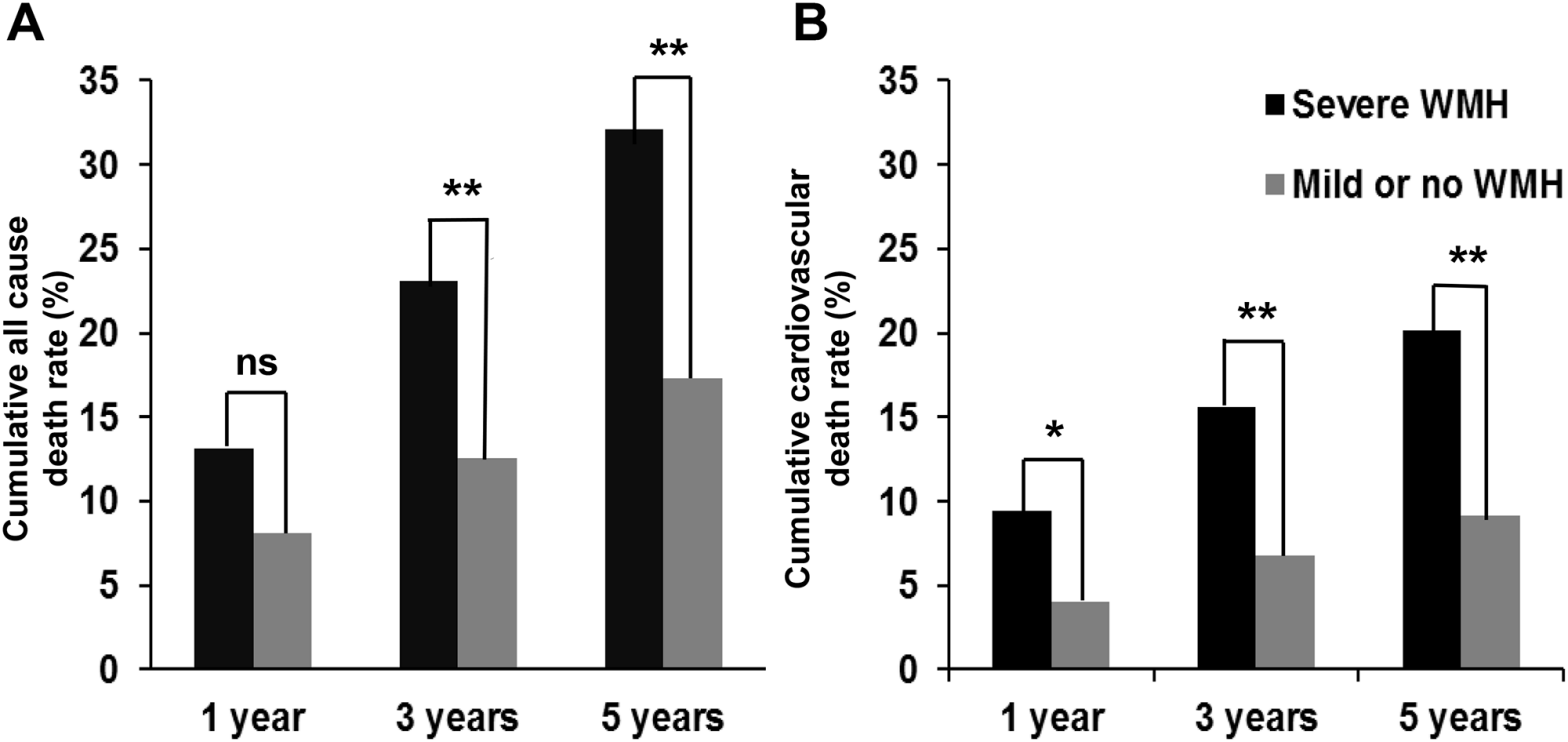
Cumulative (A) all cause and (B) cardiovascular death rate according to the presence of severe white matter hyperintensity (WMH). An asterisk indicates p <0.05; two asterisks indicates p <0.001.

Study patients were followed for a median of 7.7 years (IQR, 5.6–9.7). During the follow-up period, 200 patients (37.2%) died. Patients with severe WMH showed higher cumulative death rates compared to patients with no or mild WMH (13.2 vs. 8.1% within 1 year, p = 0.062; 23.0 vs. 12.5% within 3 years, p = 0.002; 32.1 vs. 17.3% within 5 years, p < 0.001) (Fig 1A). In total, 108 patients (20.1%) died because of cardiovascular causes: fatal stroke in 88 patients (16.4%) and fatal ischemic heart disease in 20 patients (3.7%). Patients with severe WMH showed higher cumulative cardiovascular death rates compared to patients with no or mild WMH (9.5 vs. 4.1% within 1 year, p = 0.014; 15.6 vs. 6.8% within 3 years, p = 0.001; 20.2 vs. 9.2% within 5 years, p < 0.001) (Fig 1B). All underlying causes of death including 92 patients who died because of other causes were shown in S2 Table.

### Univariable and multivariable analyses of long-term mortality

**Fig 2:**
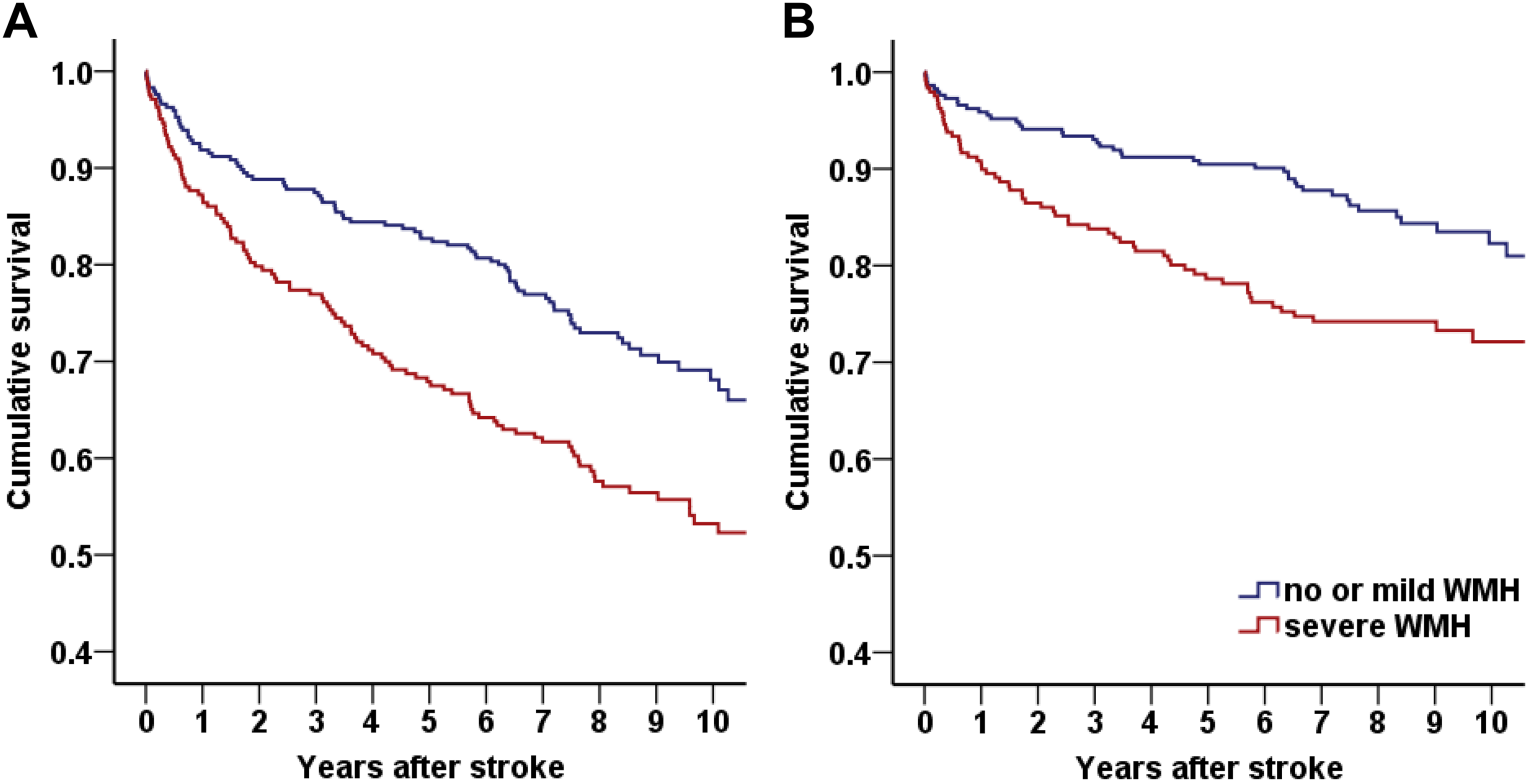
Kaplan-Meier survival curve according to the presence of severe white matter hyperintensity (WMH). Univariable Kaplan-Meier survival analysis revealed that large artery atherosclerosis (LAA) patients with severe WMH showed (A) higher mortality (p<0.001) and (B) higher cardiovascular death rate (p = 0.003) than patients with no or mild WMH.

Kaplan-Meier survival analysis showed that patients with severe WMH had a higher all-cause (p < 0.001, Fig 2A) and cardiovascular (p = 0.003, Fig 2B) death rate than patients without severe WMH. Cox regression analysis revealed that older age, history of diabetes, serum creatinine level, initial stroke severity, and the presence of severe WMH were independent predictors of long-term mortality. Patients with severe WMH had a higher death rate compared to patients with no or mild WMH after adjustment (HR 1.50, 95% CI 1.12–2.00, p = 0.007) (Table 3).

**Table 3:** Cox regression analysis of long-term mortality.

	Univariable		Multivariable	
	HR (95% CI)	*p*-value	HR (95% CI)	*p*-value
Age (years)				
<65	1		1	
≥65	2.96 (2.15–4.10)	<0.001	2.91 (2.05–4.12)	<0.001
Sex (men)	1.26 (0.93–1.72)	0.141	0.84 (0.61–1.15)	0.836
Hypertension	0.90 (0.65–1.24)	0.504		
Diabetes	1.30 (0.98–1.71)	0.067	1.48 (1.11–1.97)	0.007
Hyperlipidemia	0.84 (0.54–1.31)	0.450		
Smoking	1.00 (0.76–1.32)	0.984		
Pulse pressure (mmHg)	1.00 (0.99–1.62)	0.368		
Total cholesterol (mmol/L)	0.99 (0.85–1.14)	0.847		
Triglyceride (mmol/L)	0.78 (0.64–0.96)	0.017	0.92 (0.76–1.12)	0.397
HDL (mmol/L)	1.19 (0.85–1.65)	0.317		
LDL (mmol/L)	1.02 (0.89–1.17)	0.761		
Serum creatinine (μmol/L)	1.50 (1.33–1.69)	<0.001	1.37 (1.21–1.56)	<0.001
Initial NIHSS score				
0–2	1		1	
3–5	1.85 (1.25–2.75)	0.002	2.15 (1.43–3.23)	<0.001
≥6	2.73 (1.93–3.87)	<0.001	3.14 (2.18–4.52)	<0.001
Previous antithrombotics	1.09 (0.62–1.91)	0.771		
Previous statin	0.67 (0.27–1.62)	0.368		
Severe WMH	1.65 (1.25–2.18)	<0.001	1.50 (1.12–2.00)	0.007

Data are expressed as the hazard ratio (95% CI);

HDL, high density lipoprotein; LDL, Low density lipoprotein; NIHSS, National Institute of Health Stroke Scale; WMH, white matter hyperintensity.

### Subgroup analysis of long-term mortality according to age

**Fig 3:**
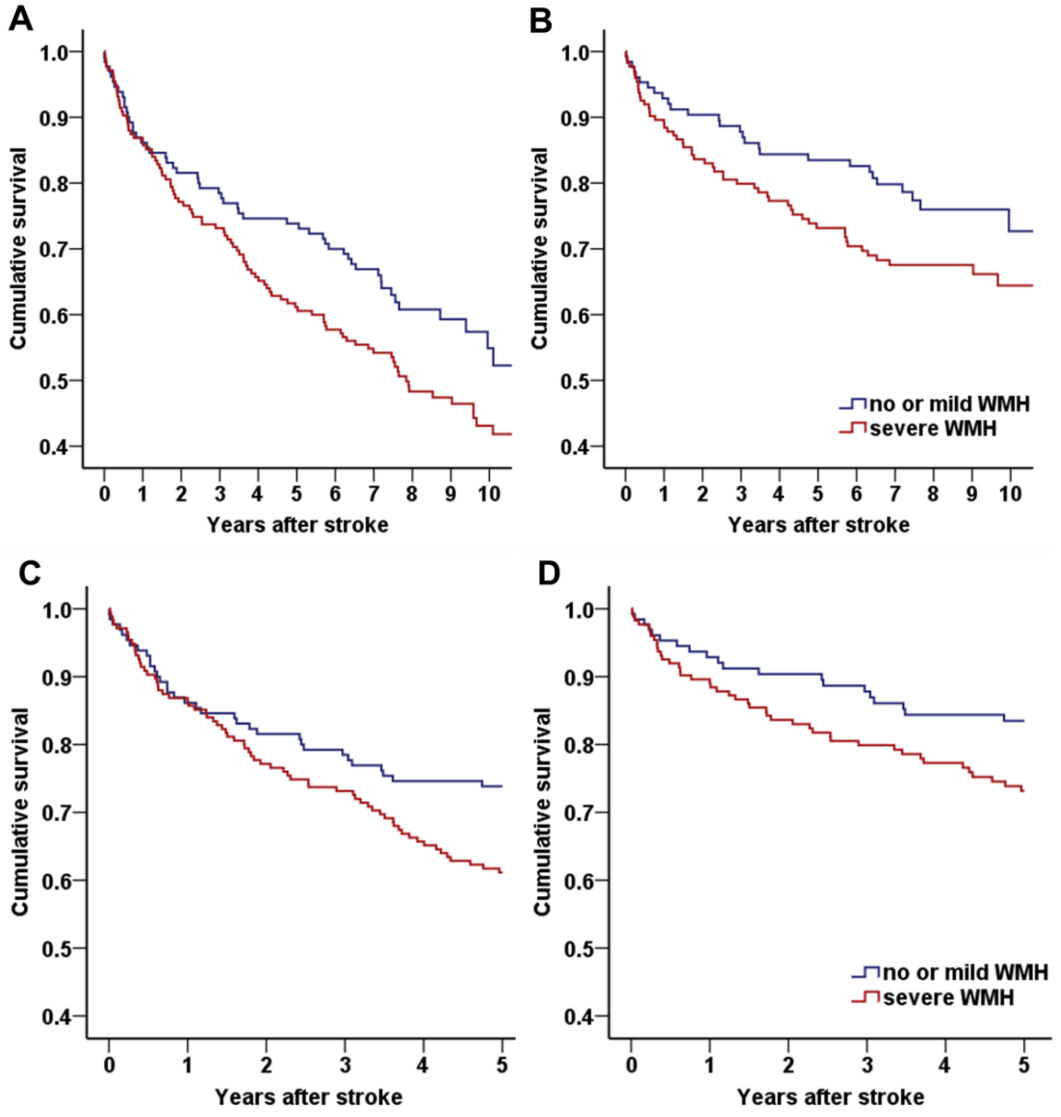
Kaplan-Meier survival curve according to the presence of severe white matter hyperintensity (WMH) in the older age group. In the older age group (age ≥65 years), patients with severe WMH showed a tendency toward a higher (A) all cause (p = 0.059) and (B) cardiovascular (p = 0.087) death rate. This association was not found in patients in the younger age group. In the limited analysis of follow-up within 5 years, older age patients with severe WMH showed significantly higher (C) all cause (p = 0.034) and (D) cardiovascular (p = 0.045) mortality rates than patients with no or mild WMH.

WMH is strongly associated with age [5], which is one of important determinants of long-term outcome. We also found that there was an interaction between age and severe WMH (unstandardized beta = 0.031, SE = 0.018, p for interaction = 0.096) in multivariable interaction model and performed additional subgroup analysis according to median age: a younger age group (<65 years old) and an older age group (≥65 years old). Patients in the older age group were more likely to have severe WMH compared to the younger age group patients (57.5% vs. 29.2%, p < 0.001). The older age group also had a higher cumulative death rate than the younger age group during follow-up (57.4 vs. 29.2%, p < 0.001). Among the older age group, patients with severe WMH showed a tendency toward a higher all cause (p = 0.059, Fig 3A) and cardiovascular (p = 0.087, Fig 3B) mortality than patients with no or mild WMH. In the younger age group, all cause and cardiovascular mortality were not different between patients with and without severe WMH (p = 0.882 and p = 0.728).

Because of the long-term follow-up period and since old age itself is a major determinant of mortality rate, we performed an additional analysis which limited the follow-up period to 5 years. The older age group with severe WMH showed higher all cause (p = 0.034, Fig 3C) and cardiovascular (p = 0.045, Fig 3D) death rates compared to patients with no or mild WMH. Cox regression revealed that older age group patients with severe WMH had a 1.75-fold (95% CI, 1.15–2.65, p = 0.008) higher 5-year mortality rate compared to patients without severe WMH (Table 4). In the younger age group, 5-year mortality was not different between patients with and without severe WMH in multivariate model (HR 1.72, 95% CI 0.78–3.82, p = 0.182).

**Table 4:** Cox regression analysis of 5-year mortality in the older age group.

	Univariable		Multivariable	
	HR (95% CI)	*p*-value	HR (95% CI)	*p*-value
Sex (men)	1.05 (0.70–1.57)	0.833	1.13 (0.74–1.71)	0.573
Hypertension	1.00 (0.62–1.61)	0.983		
Diabetes	1.07 (0.72–1.58)	0.750		
Hyperlipidemia	0.72 (0.37–1.44)	0.356		
Smoking	1.30 (0.88–1.91)	0.189		
Pulse pressure (mmHg)	1.00 (0.99–1.01)	0.555		
Total cholesterol (mmol/L)	0.97 (0.79–1.20)	0.805		
Triglyceride (mmol/L)	0.67 (0.48–0.93)	0.018	0.79 (0.58–1.10)	0.160
HDL (mmol/L)	0.90 (0.53–1.53)	0.699		
LDL (mmol/L)	1.00 (0.82–1.22)	0.979		
Serum creatinine (μmol/L)	1.39 (1.20–1.61)	<0.001	1.30 (1.12–1.50)	<0.001
Initial NIHSS score				
0–2	1		1	
3–5	2.91 (1.53–5.52)	0.001	3.59 (1.86–6.95)	<0.001
≥6	5.14 (2.92–9.04)	<0.001	5.36 (2.97–9.66)	<0.001
Previous antithrombotics	0.83 (0.39–1.80)	0.644		
Previous statin	0.88 (0.32–2.39)	0.799		
Severe WMH	1.55 (1.03–2.35)	0.036	1.75 (1.15–2.65)	0.008

Data are expressed as the hazard ratio (95% CI);

HDL, high density lipoprotein; LDL, Low density lipoprotein; NIHSS, National Institute of Health Stroke Scale; WMH, white matter hyperintensity.

### Patterns of deep and periventricular WMH

We investigated the patterns of periventricular WMH and deep WMH because the different patterns may explain the differences in pathophysiology and age effects. The coefficient of correlation between age and periventricular WMH (unadjusted ρ = 0.344, p < 0.001; adjusted ρ = 0.322, p < 0.001) was higher than that between age and deep WMH (unadjusted ρ = 0.273, p < 0.001; adjusted ρ = 0.250, p < 0.001). Steiger’s Z-test showed correlation between age and periventricular WMH were higher than that of deep WMH (unadjusted p = 0.022; adjusted p = 0.023). Multivariable Cox analyses showed that severe periventricular WMH was an independent predictor of long term mortality in all age group (OR 1.49, 95% CI 1.04–2.16, p = 0.032) and older age group (OR 1.77, 95% CI 1.18–2.66, p = 0.006). In contrast, severe deep WMH was not (p = 0.974 in all age group). In younger age group, neither severe periventricular nor deep WMH was associated with long-term mortality (p = 0.574 with severe periventricular WMH; p = 0.111 with severe deep WMH) (S3 Table).

## Discussion

This study demonstrated that severe WMH was independently associated with long-term all cause and cardiovascular mortality in stroke patients with the LAA subtype, especially in older age patients. The relationship between severe WMH and long-term mortality in patients with LAA remained significant after adjustment for well-known predictors including age and initial stroke severity.

We found that severe WMH was frequently found in patients who had LAA (45.2%). It is well known that lacunar infarction resulted from small artery occlusion is highly associated with WMH. Besides small artery occlusion, our study supported the previous report that the patients with LAA also frequently had WMH (55.4%) [7]. The association between LAA and severe WMH may be explained by reduced cerebral perfusion and cerebrovascular reactivity due to large artery atherosclerosis. A previous study reported that a higher burden of WMH was found in patients with a higher degree of carotid stenosis [8]. On the other hands, arterial stiffness may explain the association between LAA and severe WMH [9]. Previous study showed that increased arterial stiffness is strongly associated with large artery atherosclerosis at various arterial trees [14]. Arterial stiffness increases the pulse pressure and contributes to systemic vessel wall injury. Because cerebral small vessels have a low vascular resistance and are vulnerable to these increased pulse pressure, heightened arterial stiffness accompanied by LAA may be linked with WMH [9].

This study showed that severe WMH was independently associated with long-term mortality. The underlying mechanism cannot be solely drawn from our study. However, considering the association between LAA and WMH [6–8], severe WMH may be a surrogate marker of atherosclerotic burden. Higher atherosclerotic burdens in multiple vascular beds are known to be associated with poor prognosis in stroke patients [15]. In addition, increased arterial stiffness itself is an independent predictor of long-term mortality [16]. Further prospective study including evaluation of atherosclerosis in multiple vascular beds and measurements of arterial stiffness might be needed to elucidate the mechanism of the association between severe WMH and long-term mortality in patients with LAA.

In our study, functional outcome at 3 months was similar regardless of the presence of severe WMH. In a longitudinal cohort study of all subtypes of ischemic stroke, those with severe WMH showed poor functional outcomes at 3 months [17]. On the other hand, because WMH is a long-term process, short-term outcomes may not be related with the presence of severe WMH. In addition, because the overall prognosis of patients with LAA subtypes is poor among stroke subtypes, short-term outcome may be encompassed with poor prognosis of the LAA subtype [18]. In contrast, in terms of long-term mortality, the burdens of both large and small cerebral arteries might have a synergistic effect on long-term prognosis.

In subgroup analyses, we found that older age in patients with severe WMH was associated with higher mortality within 5 years. In contrast, there was no such association in younger patients. Our finding suggests that the impact WMH on long-term outcome is different according to age in stroke patients with LAA. Both age and presence of WMH may have an impact on the long-term prognosis. However, our results may be confounded from relative small number of younger age group. To confirm this, further large studies might be needed.

Among the WMH, we found that older age group was highly associated with periventricular WMH than deep WMH. For an increase in each age decade, periventricular WMH showed a greater volume increase than deep WMH [19]. The periventricular white matter is located in the arterial border zone and supplied by long perforating arteries which makes it particularly vulnerable to decreases in cerebral blood flow [8]. A previous report showed that severe periventricular WMH increased the risk of death, while deep WMH did not [20]. In addition, another study showed that cardiovascular risk factors were associated with periventricular WMH, but not with deep WMH [21]. In our study, severe WMH, especially periventricular, was associated with higher mortality only in the older age group (S3 Table). Therefore, our findings of differential impact of severe WMH on long-term outcome according to age may be explained by the higher involvement of periventricular white matter in the older age group compared to the younger age group.

There were some limitations in our study. First, this study is a retrospective analysis in a single stroke center, so possible selection bias may exist. However, the enrollment of consecutive patients and long-term follow-up are strengths of our study. We conducted long-term follow-up of patients for over 7 years. Because WMH is a chronic process, the impact of WMH on mortality is difficult to determine with a short follow-up period. Second, we did not quantify WMH burden with volumetry, which has been reported as more sensitive and can help avoid the ceiling-effect of ordinal scales. However, the Fazekas scale is well established and was found to be well correlated with WMH volume in a previous study [22]. Third, although we enrolled the patients with LAA, subclinical cardiac dysfunction could be one of confounding factor [23]. In this study, echocardiography was performed in only about half of patients. Further prospective study, which evaluates echocardiography in all study patients might be helpful.

## Conclusion

Severe WMH was independently associated with long-term mortality in stroke patients with the large artery atherosclerosis, especially in the older age group. Burdens of atherosclerosis in both small and large arteries might have a synergistic impact on the poor long-term prognosis in ischemic stroke patients.

## Supporting information

S1 Table Etiologic evaluations according to degree of WMH. WMH indicates white matter hyperintensities; CTA, CT angiography; MRA, MR angiography; DSA, digital subtraction angiography; TEE, transesophageal echocardiography; TTE, transthoracic echocardiography. Values are n (%).
(DOCX)

S2 Table Causes of death. (DOCX)

S3 Table Cox regression analysis of long-term mortality in patients with severe periventricular or deep WMH according to age group. * Adjusted for sex and the variables with p<0.1 in univariable analyses. WMH, white matter hyperintensity.
(DOCX)
