# Supplementary material for: Differential impact of white matter hyperintensities on long-term outcomes in ischemic stroke patients with large artery atherosclerosis
Source: PLoS One. 2017 Dec 12;12(12):e0189611. doi: 10.1371/journal.pone.0189611 (PMC5726763; doi:10.1371/journal.pone.0189611)
Supplement: S1 Table — WMH indicates white matter hyperintensities; CTA, CT angiography; MRA, MR angiography; DSA, digital subtraction angiography; TEE, transesophageal echocardiography; TTE, transthoracic echocardiography. Values are n (%). (DOCX) [file pone.0189611.s001.docx]

**S1 Table. Etiologic evaluations according to degree of WMH**

|  | Total  (n = 538) | No or mild WMH  (n = 295) | Severe WMH  (n = 243) |
| --- | --- | --- | --- |
| Angiographic evaluations | 538 (100) | 295 (100) | 243 (100) |
| CTA | 83 (15.4) | 47 (15.9) | 36 (14.8) |
| MRA | 448 (83.3) | 237 (80.3) | 211 (86.8) |
| DSA | 241 (44.8) | 155 (52.5) | 86 (35.4) |
| Cardiac image | 262 (48.7) | 164 (55.6) | 98 (40.3) |
| Heart CT | 54 (10.0) | 41 (13.9) | 13 (5.3) |
| Echocardiography | 246 (45.7) | 153 (51.9) | 93 (38.3) |
| TEE | 219 (40.7) | 139 (47.1) | 80 (32.9) |
| TTE | 38 (7.1) | 21 (7.1) | 17 (7.0) |
| Continuous EKG monitoring | 388 (72.1) | 208 (70.5) | 180 (74.1) |

WMH indicates white matter hyperintensities; CTA, CT angiography; MRA, MR angiography; DSA, digital subtraction angiography; TEE, transesophageal echocardiography; TTE, transthoracic echocardiography.

Values are n (%).
