# Supplementary material for: Differential impact of white matter hyperintensities on long-term outcomes in ischemic stroke patients with large artery atherosclerosis
Source: PLoS One. 2017 Dec 12;12(12):e0189611. doi: 10.1371/journal.pone.0189611 (PMC5726763; doi:10.1371/journal.pone.0189611)
Supplement: S2 Table — (DOCX) [file pone.0189611.s002.docx]

**S2 Table. Causes of death**

| Underlying causes of death | Number of deaths | Percentage  of all deaths |
| --- | --- | --- |
| Fatal stroke | 88 | 44.0 |
| Fatal ischemic heart disease | 20 | 10.0 |
| Malignant neoplasms | 39 | 19.5 |
| Endocrine disorders | 16 | 8.0 |
| Respiratory disorders | 8 | 4.0 |
| Digestive disorders | 6 | 3.0 |
| Infectious disorders | 5 | 2.5 |
| Other medical disorders | 4 | 2.0 |
| Other circulatory disorders | 3 | 1.5 |
| Injury, poisoning and other consequence of external causes | 6 | 3.0 |
| Unspecified causes | 5 | 2.5 |
