# Supplementary material for: Differential impact of white matter hyperintensities on long-term outcomes in ischemic stroke patients with large artery atherosclerosis
Source: PLoS One. 2017 Dec 12;12(12):e0189611. doi: 10.1371/journal.pone.0189611 (PMC5726763; doi:10.1371/journal.pone.0189611)
Supplement: S3 Table — * Adjusted for sex and the variables with p<0.1 in univariable analyses. WMH, white matter hyperintensity. (DOCX) [file pone.0189611.s003.docx]

S3 Table**. Cox regression analysis of long-term mortality in patients with severe periventricular or deep WMH according to age group**

|  | All age | | Age ≥ 65 | | | Age < 65 | |
| --- | --- | --- | --- | --- | --- | --- | --- |
|  | HR (95% CI) | *p*-value | | HR (95% CI) | *p*-value | HR (95% CI) | *p*-value |
| Severe periventricular WMH | 1.49 (1.04-2.16) | 0.032 | | 1.77 (1.18-2.66) | 0.006 | 0.76 (0.30-1.96) | 0.574 |
| Severe deep WMH | 1.01 (0.69-1.47) | 0.974 | | 0.87 (0.58-1.30) | 0.496 | 2.31 (0.83-6.47) | 0.111 |

* Adjusted for sex and the variables with p<0.1 in univariate analyses

WMH, white matter hyperintensity.
